# Knowledge, Hygienic Practices, and Toxi-Infectious Risks Associated with Ready-to-Eat Gbeli: A Particular Chip Derived from Cassava (*Manihot esculenta* Crantz) Tuber Vended in Streets of Abomey-Calavi Municipality, Benin

**DOI:** 10.1155/2022/8399831

**Published:** 2022-11-17

**Authors:** Agossou D. P. Noumavo, Messan A. B. Ohin, Ibilola G. Fadipe, Bruno Hadji, Sébastien Ahouangansi, Yanik Y. Akin, Lamine Baba-Moussa, Farid Baba-Moussa

**Affiliations:** ^1^Laboratoire de Microbiologie et de Technologies Alimentaires, Département de Biologie Végétale, Faculté des Sciences et Techniques, Université d'Abomey-Calavi, Abomey-Calavi, 04 BP 1107 Cotonou, Benin; ^2^Laboratoire de Biologie et de Typage Moléculaire en Microbiologie, Département de Biochimie et de Biologie Cellulaire, Faculté des Sciences et Techniques, Université d'Abomey-Calavi, Abomey-Calavi, 05 BP 1604 Cotonou, Benin; ^3^Laboratoire de Biomathématiques et d'Estimations Forestières, Faculté des Sciences Agronomiques, Université d'Abomey-Calavi, Abomey-Calavi, 04, BP 1525 Cotonou, Benin

## Abstract

The lack of regulations relating to street food remains a real problem in most developing countries. To remedy this, it is essential to have reliable data on the actors and the conditions of streed food activity. The present study is aimed at (i) establishing the sociodemographic profile of producers and vendors of Gbeli in the municipality of Abomey-Calavi, (ii) developing the technical production diagram and assessing the adoption level of good hygienic practices during the production and sale of Gbeli, and finally, (iv) evaluating the microbiological contamination risks associated with Gbeli consumption. Direct observations followed by a semistructured survey on 92 Gbeli vendors were carried out in Abomey-Calavi municipality. A Gbeli sample was collected from each vendor for microbiological analyses according to standard microbiology analytical techniques. Forty vendors were assisted during Gbeli production. The results showed that the production and sale of Gbeli are activities carried out solely by women (100%), mostly adults (97.82%) and unschooled (58.69%). This trade generates income allowing the vendors to contribute to their household expenses and to save. No major difference was noted in the production process of Gbeli contrary to the sale modes (stationary, itinerant, and mixed). Production process, packaging form, and sale condition of Gbeli present enormous hygienic practice deficiencies. Some corrective measures were thus suggested. About 56% of Gbeli samples analyzed were microbiologically not up to standard. The prevalence and abundance of mycotoxinogenic molds, thermotolerant coliforms, and coagulase-positive *Staphylococci* in these chips are very worrying. The consumption of Gbeli therefore exposes its consumers to a permanent risk of food poisoning. Training of Gbeli vendors on good hygiene and production practices is indispensable.

## 1. Introduction

Street catering is an ancient practice common to several countries. It is on rise in the main cities of most developing countries. Street foods from this catering mode are defined as ready-to-eat foods, prepared and/or sold especially in streets and public places [1]. They allow certain social groups (civil servants, pupils, students, craftsmen, tradesmen, etc.) to eat inexpensively outside their homes. It contributes significantly to the nutrition and food security of many populations with low and modest incomes [2]. Street food businesses contribute significantly to income generation for many people from low-income households involved in this activity [3]. It is one of the main ways to preserve the great culinary diversity of developing countries. These foods are prioritized by consumers because of their low cost, availability, and unique taste related to the cultural and social values of the region.

Unfortunately, street food sector is mostly informal and poorly regulated in developing countries [4]. The informal nature of street food sector associated with the lack of basic infrastructure (electricity, drinking water, refrigerator, storage facilities, food reheating equipment, waste disposal facilities, etc.) compromises the sanitary quality of meals. This situation contributes to meal deterioration and exposes street food consumers to permanent risk of food poisoning. Street foods can be a source of disease due to glaring lack of hygiene practices throughout the production chain [5]. These foodborne diseases are caused by the consumption of food contaminated with pathogenic microorganisms and/or their toxins [6]. Pathogenic microorganism growth is accelerated by the often inadequate preservation methods. In addition, the street food producers and vendors lack information on food safety and foodborne diseases. The World Health Organization (WHO) has reported that 420,000 people die each year from foodborne illness [7]. Africa and Asia have the highest mortality rates from foodborne diseases [8]. Also, the financial resources using to treat diseases caused by street food consumption impoverish both populations and states.

Faced of this public health problem, the developing countries (in particular Africa) must develop control and monitoring programs for actors of street food sector. Achieving this objective requires a better knowledge of the sector. It is in this context that the present study based on Gbeli was initiated. Gbeli is a particular chip formulated from cassava (*Manihot esculenta* Crantz) tuber. It is highly prized in the municipality of Abomey-Calavi (Benin), especially by craftsmen, schoolchildrens, and students. This study is aimed at (i) establishing sociodemographic characteristics of Gbeli vendors and incomes, (ii) elaborating process diagram for Gbeli production and evaluating hygienic practice level during both production and sale, and (iii) assessing the toxi-infectious risks associated with Gbeli through microbiological quality evaluation.

## 2. Materials and Methods

### 2.1. Geographical Area

**Figure 1:**
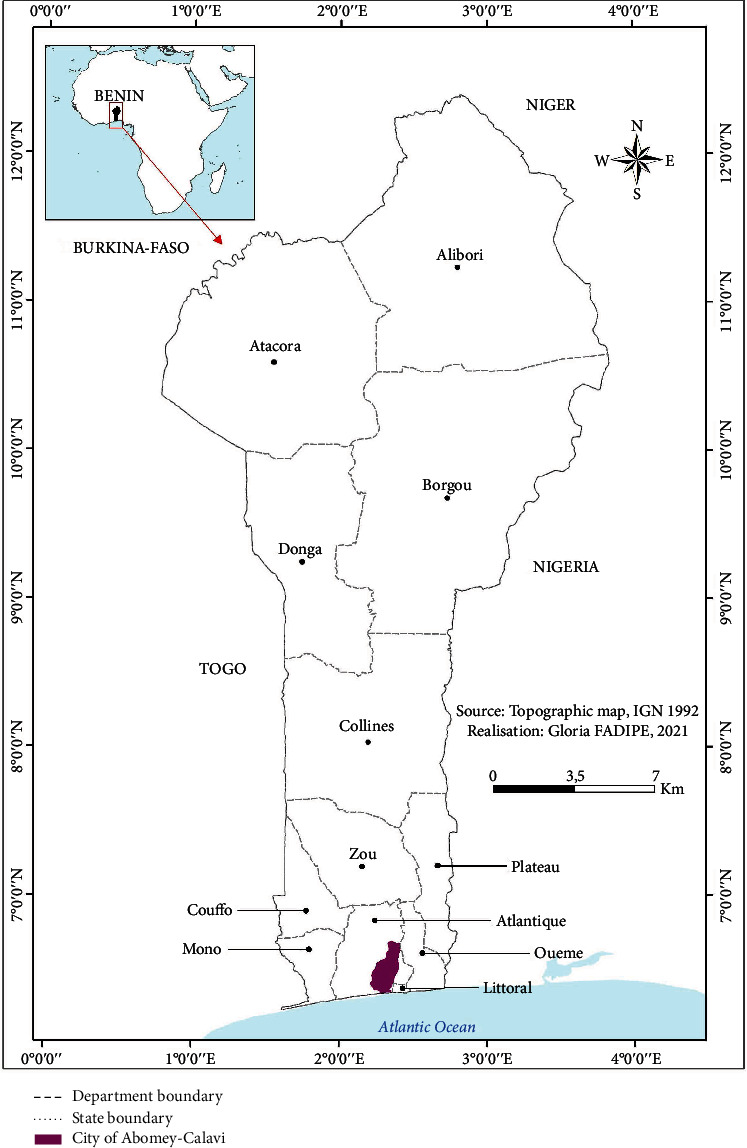
Geographical location of study area.

This study was carried out in Abomey-Calavi City (Figure 1) located in Atlantic department, the most populous department of Benin republic. It is the largest city of this department. This dormitory city was chosen due to its high population (656358 people), population density (1010 people/km^2^) [9], and concentration of Gbeli street food vendors.

### 2.2. Study Population

This study was carried out during the period of April to May 2018. The population considered in this study is comprised of producers and vendors of ready-to-eat Gbeli. A total of ninety-two (92) producers and vendors were randomly selected and gave their consent to participate in this study. The methodological approach adopted consisted of making direct observations followed by a semistructured survey based on a preestablished questionnaire. Before administering the questionnaire, a brief presentation of the study context and questionnaire content was orally explained to respondents (producers and vendors of Gbeli) face-to-face in the local dialect understood by them. A confidentiality agreement has been concluded with the respondents.

### 2.3. Questionnaire Design

The questions contained in the questionnaire are aimed firstly at establishing the sociodemographic profile of the producers and vendors of ready-to-eat Gbeli. Secondly, assess the impact of this business on family financial health of producers and vendors. Thirdly, develop the technological diagram of Gbeli production, and finally, assess the adoption level of hygienic standards by these producers and vendors. The questionnaire is structured in five sections. The first section regarded sociodemographic and professional training characteristics of respondents (sex, age, instruction level, professional formation, and membership of food vendor association). The second and third sections, respectively, contained questions relating to knowledge and impact of this business on family financial level. The fourth section was related to type of seller, while the last section was related to hygiene and production conditions (source of water, kind of packaging materials, personal hygiene of vendors, environmental sanitation, etc.). It should be noted that forty (40) producers were assisted during Gbeli production in your industrial units in order to identify all steps and conditions of the production. After each interview step, Gbeli ready-to-eat (100 g) were aseptically sampled from vendors at the point of sale. Each sample was placed in the sterile bag, closed immediately, kept in a cooler containing ice, and sent directly to the laboratory for microbiological quality assessment.

### 2.4. Microbiological Analysis

Microbiological quality assessment of the ready-to-eat Gbeli samples was performed according to the standard methods used by Ohin et al. [10] with few modifications. It consisted in the enumeration of total mesophilic aerobic microorganisms on Plate Count Agar (Oxoid, England) after incubation at 30°C for 72 h. Total coliforms and fecal coliforms were isolated on Violet Red Bile Lactose Agar (Liofilchem Diagnostici, Italy) after incubation, respectively, at 30°C and 44°C for 24 h. Coagulase-positive *Staphylococci* were isolated on the Baird-Parker Agar (Biokar Diagnostics, France) enriched with egg yolk and potassium tellurite following incubation at 37°C for 24 h to 48 h. Yeasts and molds were enumerated on the Sabouraud Agar (Biokar Diagnostics, France) supplemented with chloramphenicol (25°C for 5 days). Tryptone Sulfite Neomycin Agar (Biokar Diagnostics, France) was used to enumerate anaerobic sulfite reducing (ASR) bacteria under strict anaerobic conditions (44°C for 24 h). After preenrichment in Buffered Peptone Water (HiMedia, India) following the enrichment in Rappaport-Vassiliadis Selective Broth (Oxoid, United Kingdom) both incubated at 37°C for 24 h, *Salmonella* spp. were isolated on selective media Xylose Lysine Deoxycholate Agar (Oxoid, United Kingdom) and Brilliant Green Agar (Oxoid, England) both incubated at 37°C for 24 h. It should be noted that there are no local microbiological standards for Gbeli and similar products. We referred to chips microbiological criteria of Luxembourg [11].

### 2.5. Data Management and Analysis

Data from the survey forms were encoded using the Microsoft Excel 2013 and analyzed using R software version 4.0.0. The data were subjected to descriptive statistical tests (proportion, mean, and standard deviation). The microbiological parameters evaluated were submitted to analysis of variance (probability level of 5%), following a mean separation (Student-Newman-Keuls test). A principal component analysis to describe the relationship between the abundance of different types of microorganisms was also performed.

## 3. Results and Discussion

### 3.1. Sociodemographic Characteristics of Gbeli Vendors

Sociodemographic characterization assessment of Gbeli vendors reveals that only women sell Gbeli (100%) in the municipality of Abomey-Calavi (Table 1). Ohin et al. [12] revealed that only women sell *Borassus aethiopum* Mart hypocotyls in the streets of Cotonou City (Benin). Several studies around the world are unanimous on the predominance of women in the street catering [12–16]. In contrast, Cortesea et al. [17] noticed a predominance of men (58%) in street catering in Florianopolis (Santa Catarina State, Brasil). Indeed, several factors can influence the gender profile in street catering (types of food, geographical areas, cultural considerations, etc.). For example, in West Africa, grilling meat is speciality of men while the preparation of meals is rather reserved for women. Other culinary specialities (curdled milk, ice cream, etc.) may be carried out by men or women equally. Ethnic specialisation is sometimes noted. For example, the Peuhl, Maure, and Haoussa are in the majority in the grilling of meat.

The majority of Gbeli vendors (60.9%) were between 30 and 39 years old (Table 1). Only one vendor was under 20 years old. This young girl was occasionally called upon by her mother who was ill. Indeed, street catering food is quite often a family business to which minors and adolescents contribute in Africa. Food and Agriculture Organization of the United Nations revealed that the average age of street food vendors was 35 years old [16]. Twenty-seven years later, the trend has not changed according to the results obtained in this study. Drabo et al. [15] found that the majority (60.9%) of street food vendors in Bobo-Dioulasso (Burkina Faso) were between 20 and 39 years old. This age group to which most street food vendors belong in developing countries (especially in sub-Saharan Africa) can be explained by several reasons. Between 25 and 38 years old, the majority of women and men are in couples with children. They need money to support the family. They therefore engage in any activity that can bring them a regular income. Street food sector suits them well because it does not require large investments at the beginning.

It was noted during this study that the majority of Gbeli vendors have no formal education. Almost 60% of them have never been to school. Only 23.9% have attended primary school. This result is similar to that obtained by Samapundo et al. [4] in a study carried out in Ho Chi Minh City (Vietnam). In this study, the authors noted that most street food vendors have a low education level. One of the consequences of this low education level is the lack of hygienic practice knowledge which is often the cause of food contamination [18].

### 3.2. Technical Process of Gbeli Production

**Figure 2:**
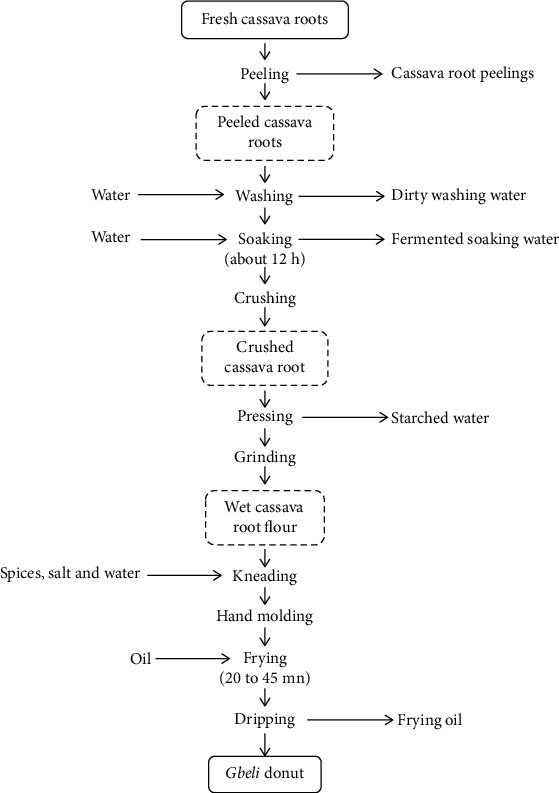
Process diagrams for Gbeli production.

Figure 2 shows the different stages of Gbeli production. First of all, it should be noted that it is the woman vendors themselves who produce the Gbeli. They are therefore both producers and vendors.

**Figure 3:**
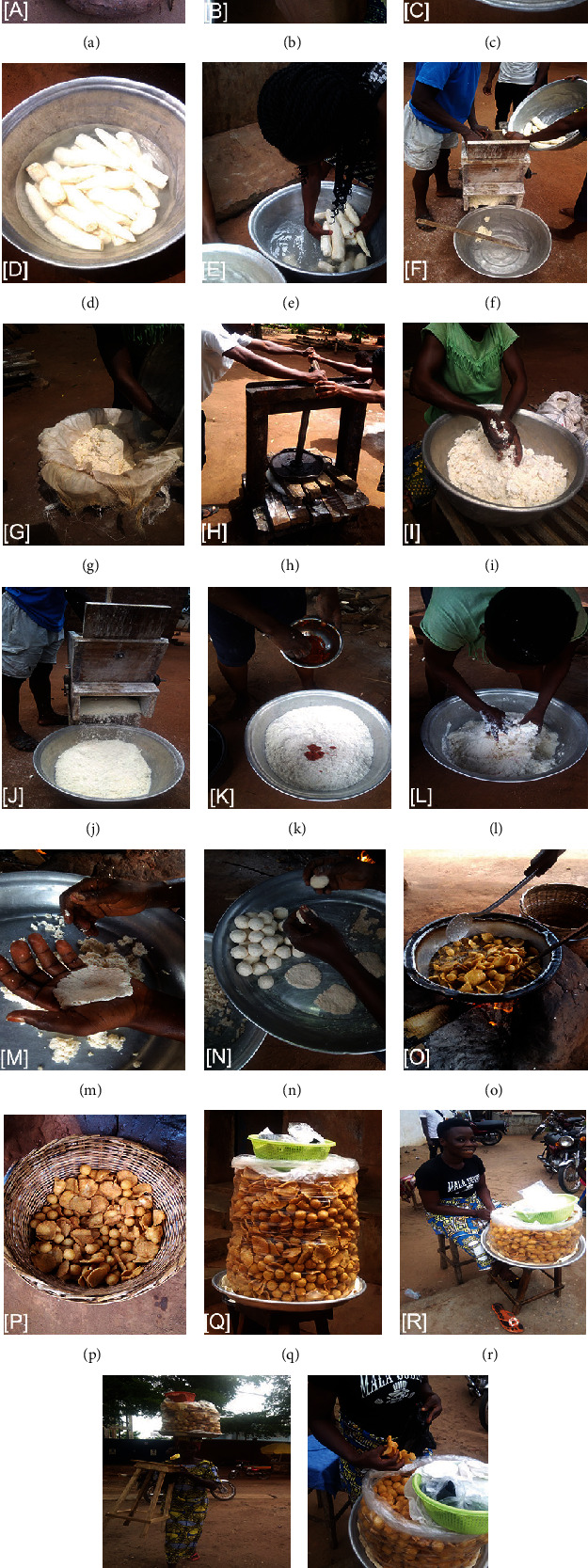
Different unit operations in the Gbeli production: (a) cassava tubers, (b) peeling, (c) washing, (d, e) soaking, (f) crushing, (g, h) pressing, (i, j) grinding, (k) adding ingredients, (l) kneading, (m, n) hand molding, (o) frying, (p) dripping, (q) packing, and (r–t) sales methods.

The Gbeli production begins with the buying of cassava tubers (Figure 3(a)) from cassava producers in Abomey-Calavi municipality and surrounding villages. Most of Gbeli producers go to the production fields to buy cassava tubers. With the familiarity that is gradually being created between Gbeli producers and cassava producers, some Gbeli producers have cassava tubers delivered to their homes. The cassava tubers are first peeled (Figure 3(b)). The peeled tubers are washed and then soaked in water (Figures 3(c) and 3(d)).

About 12 h later, the tubers are carried off from water and crushed using a traditional grinder (Figures 3(e) and 3(f)). The crushed cassava tuber obtained is pressed using a traditional press machine and ground (Figures 3(g)–3(j)). The wet cassava tuber floor obtained is added with water, salt, and spices (chili pepper, garlic, onion, ginger, etc.) and then kneaded (Figures 3(k) and 3(l)). The resulting cassava tuber flour dough is molded by the hand into the two main shapes of Gbeli (round and flat) and fry for an average of 30 minutes (Figures 3(m)–3(o)). After dripping, the Gbeli are packaged for sale (Figures 3(p)–3(t)).

### 3.3. Mode of Gbeli Sale and Treatment of Unsold Products

Only one packaging method is used by the Gbeli vendors. The Gbeli are carefully placed in a large transparent white plastic bag (nonbiodegradable) on a tray. This bag is covered with a cloth to ventilate the product and facilitates its sale (Figure 3(q)). As the display is frequently exposed to the sun, the effects of the sun's rays on this plastic packaging could compromise the health of consumers, especially as the chips contain oil. Indeed, this packaging presents chemical contamination risks with the short- or long-term harmful effects on health of Gbeli consumers. Many manufacturing additives and residual plastic monomers that have a negative impact on consumer health can migrate from plastic packaging to food product, especially liquid and fatty products [19, 20].

Three modes of sale are used by Gbeli vendors: stationary mode (34.78%), itinerant mode (34.78%), and mixed mode (30.44%). The stationary mode consists of choosing a fixed location where the display is set up for the entire selling period (Figure 3(r)). Itinerant vendors walk from street to street to sell (Figure 3(s)), while mixed mode vendors combine the two previous modes. These three sales modes are common in our country (Benin). Moussé et al. [21] and Sina et al. [22] referred to the stationary and itinerant sale modes in their studies on salad, vegetable sauce, and cooked rice sold in the municipality of Abomey-Calavi and Cotonou (Benin).

It should be noted that the daily production of Gbeli is not always sold on the same day. Thus, it is not uncommon to find women vendors stationed along the main roads late at night (11 pm) trying to sell as much as possible. When they returned at home, the majority (95%) of vendor gave the unsold goods to children and family members, while some vendors (5%) did so the next day for resale.

### 3.4. Profitability of Gbeli Trade

The trade of Gbeli is a fairly profitable income-generating activity. It should be remembered that it is the vendors themselves who produce Gbeli. Thus, when they subtract the expenses incurred in the Gbeli production from the selling price, they obtain a significant profit. Indeed, 75.5% of Gbeli vendors said that their daily income was between 3.4 USD and 5.1 USD (Table 2). The Gbeli trade allows the saleswomen to provide for the most basic needs. This daily profit is higher than the one obtained by boiled hypocotyls (*Borassus aethiopum* Mart) vendors (2 USD) in streets of Cotonou City (Benin). The profit made by Gbeli vendors is mainly used to cover certain household expenses related to housing, food, clothing, health, and school. Part of the profit is economized by traditional tontine systems. However, the funds economized are used more for social expenses (community celebrations, weddings, deaths, etc.) than for productive investment in this Gbeli trade.

The selling price of a unit of Gbeli today is 0.017 USD (round and flat form). But it should be noted that this standardisation of Gbeli selling price is contemporary. In earlier times, there were Gbeli of 0.008 USD, 0.017 USD, and 0.043 USD in different sizes. Some vendors (6.5%) said that they received orders for Gbeli. Only one (01) confided that she made home deliveries (2.2%).

### 3.5. Hygienic Deficiency Points Noted during Gbeli Production and Sale

Hygiene practice application plays an essential role in the prevention of foodborne diseases. Hygiene practices in food processing units must take into account the production environment, equipment, staff, raw material, and the production methodology. Rigorous application of hygiene practices can prevent and limit the spread of infectious agents. This chapter reviews the hygienic deficiencies noted during Gbeli production and sale in Abomey-Calavi municipality (Benin).

#### 3.5.1. Water Use

Only 6.5% of vendors use tap water for washing material, Gbeli production, and as an ingredient (Figures 3(c)–3(e) and 3(l), Table 2). The majority (93.5%) of producers use drilling water sold by private entrepreneurs. Indeed, the national drinking water company covers only a small part of national territory. The use of drilling water should not be a problem even if none (00%) of Gbeli producers treat the water before use. Unfortunately, in order to maximize their profits, the owners of drilling water stations do not regularly maintain the system, i.e., effectively wash the tanks and water canalization and water filters, which are essential for obtaining acceptable quality of water. Awareness raising of this private entrepreneurs on good maintenance practices for drilling water system is therefore necessary. Gbeli producers should also be trained in basic water treatment methods. For example, they can use water treatment by boiling. Boiling treatment is simple to implement. It kills almost all germs and microorganisms present in the water. Indeed, the water is previously filtered or decanted and then simmer for about a minute. Gbeli producers can also use the water treatment method by chlorination. Chlorination is a simple and effective way to disinfect water in order to make it drinkable. It consists of introducing chlorinated products (chlorine tablets, bleach, etc.) into the water to kill the microorganisms contained in it. After an action time of 30 minutes, the water is drinkable. It remains so for a few days (depending on storage conditions) thanks to the residual effect of chlorine.

The water used by Gbeli producers is a priori of poor quality. It is consequently a potential and permanent sources of microbiological and chemical contaminations of Gbeli. In the study conducted by Cortesea et al. [17] in Florianopolis (Brasil), the preparation and washing water was of very poor quality in almost all street food vendors. Indeed, contaminated water poses a public health risk when used as a beverage, ingredient (incorporated into food), or to wash food, equipment, utensils, and hands [23]. This poor quality water is well known to contain enteropathogenic microorganisms such as *E. coli*, *Salmonella* spp., *Campylobacter* spp., *Vibrio cholerae*, and fecal streptococci [24].

#### 3.5.2. Production Equipment

This study showed that 97.8% of Gbeli producers had equipment used only for the production and sale of this donut (Table 3) except for the grinder and press. This is a good hygienic attitude. It could limit the possible contamination risks from other foods prepared with the same equipment. It should also be noted that the majority (86.9%) of Gbeli producers wash their production equipment twice a day (before and after production). Even though the water used is often poor quality, this attitude is to be encouraged. The production equipment is also a potential source of food contamination. The defective quality of production equipment associated with its poor hygienic use can promote the proliferation of pathogens and their toxins production but also food recontamination [23]. Also, poor equipment maintenance can cause food residues accumulation, facilitating microbial growth and thus a high contamination probability during the production process. Proper use of equipment and utensils is therefore important to prevent cross-contamination.

#### 3.5.3. Production and Sales Environment

8.7% of Gbeli producers surveyed produce outdoors sometimes under trees. The majority (73.9%) have kitchens with straw roofs (Table 3). These open-sided kitchens are made only of pillars and roofs exposing Gbeli production to atmospheric contaminations (drought, dust, etc.). Debris from straws and trees are potential contaminants. Alimi [2] had noted that most street food is usually prepared in bulk. Despite this, almost all (88%) of Gbeli production units visited are maintained in an acceptable sanitary condition (Table 3). The sale sites are also in an acceptable sanitary condition, even though Gbeli are exposed to various contaminants from the drought, dust, exhaust fumes, etc. Indeed, Gbeli vendors do not have a sale shed. They install in the proximity of public roads, institutions, churches, schools, etc. (Figures 3(r) and 3(s)). Sukontason et al. [25] reported that food placed near the ground and exposed to dust has been associated with foodborne illnesses such as cholera and diarrhea.

#### 3.5.4. Packaging and Sale

The Gbeli packaging method for sale does not respect the guidelines of food hygienic standards. The chips are placed in a large transparent nonbiodegradable plastic bag on a tray (Figure 3(q)). This plastic bag is covered with a cloth on the surface to allow air ventilation and facilitate its sale. This cloth is partially removed each time the vendor wants to serve a consumer. Mosupye and von Holy [26] reported that ready-to-eat foods can be left uncovered for up to 10 minutes when vendors serve other consumer. This packaging and selling method exposes Gbeli to a many atmospheric contaminations (Figures 3(r) and 3(s)). It would be more hygienic to package the Gbeli in a clear glass box for sale.

#### 3.5.5. Use of Bare Hands during Sale

During the sale, the majority of Gbeli vendors use the bare hands to sell (Figure 3(t)). They should use glove, fork, or spoon. This behavior exposes customer to high risk of microbiological contamination because the chips are directly consumed and there is no further step to eliminate the danger. Indeed, the same hands used by vendors to serve the Gbeli are used to take money and give change to the customers. Some vendors handle their mobile phones or wave to customers or friends during the sale. All these practices increase the risk of severe foodborne illness. The hands, banknotes, and coins are real transmission vectors of pathogenic microorganisms. A comparative study on the risks of using hands to serve street food in Ghana by Mensah et al. [27] showed that using bare hands to serve increases the contamination level. Enteropathogenic microorganisms such as *Salmonella typhi* that can survive on the human hands for many hours were isolated from street food vendors' hands by previous authors. Pathogenic *E. coli* strains usually isolated from diarrhoeal stools have been isolated from some street food vendors' hands in Thailand [28]. To serve Gbeli to customers, vendors must use a fork or wear gloves.

#### 3.5.6. Packaging and Sale Duration

Gbeli sale usually starts at around 1 pm and lasts until around 8 pm. Some vendors sell until late in the night (11 pm). Gbeli is therefore kept at room temperature for several hours, usually under the sun. This sale condition is very favorable to mesophilic microorganisms proliferation. Alimi [2] found very high numbers of aerobic pathogenic bacteria in salads and fruit juices sold at room temperature on Johannesburg streets (South Africa), despite being cooked before sale. The majority of street food vendors in Abeokuta, Nigeria (90%), and Ozamiz city, Philippines (55%), prepare food in the morning and store it at room temperature to sell until the afternoon without reheating it [29, 30]. Nonregulation of food holding time and temperature had been recognized as major risk factors in the occurrence of foodborne illness [31].

#### 3.5.7. Some Good Hygiene and Manufacturing Practices to Recommend to Gbeli Producers/Vendors

In view of the hygienic and technical shortcomings noted during the production and sale of Gbeli, we suggest some corrective measures, summarised in Table 4.

#### 3.5.8. Microbiological Quality of Gbeli

Table 5 presents the results relating to the microbiological quality of the Gbeli samples. It allows us to estimate the toxi-infectious risks to which consumers of Gbeli are exposed. The results obtained were compared with the microbiological criteria applicable to foodstuffs, specifically chips of Luxemburg Health Ministry [11]. The results of analysis of variance show a very highly significant difference in the total aerobic mesophilic flora (TAMF) from one Gbeli sample to another (*p* < 0.001). However, the average TAMF load of Gbeli obtained in this study (3.7 × 10^3^ CFU/g) is close to those obtained by Ike et al. [33] on potato (3.2 × 10^3^ CFU/g) and plantain (2.9 × 10^3^ CFU/g) chips. Indeed, the TAMF load is a good indicator of hygiene, which makes it possible to assess microbial pollution and the general quality of a foodstuff [34]. This variation of the TAMF obtained in the present study can be explained by the fact that the adoption level of hygiene practices, packaging, and sale varies enormously from one vendor to another. Also, the level of exposure to different contaminants varies according to the sale mode (stationary, itinerant, and mixed).

The average fungal load (7.4 × 10^3^ CFU/g) of the Gbeli samples is higher than those obtained by Ike et al. [33] on potato chips (1.2 × 10^2^ CFU/g) and plantain chips (0.7 × 10^2^ CFU/g). The presence of molds and yeasts in a food should not be ignored. Indeed, many of them, in particular mycotoxinogenic molds (aflatoxins, ochratoxins, and fumonisins production), can harm human health when ingested in high concentrations. These mycotoxins have been reported to be carcinogenic, hepatotoxic, immunosuppressive, and embryotoxic [35].

In this study, it was also noticed that the Gbeli samples contain germ indicators of fecal contamination such as coliforms. The average thermotolerant coliform load (1.4 × 10^2^ CFU/g) is slightly higher than the standard value (10^2^ CFU/g). About twenty-one percent (21%) of the Gbeli samples had a thermotolerant coliform load above the threshold tolerated in chips (Table 5). Among the thermotolerant coliforms, the *Escherichia coli* species is predominant. Indeed, *E. coli* is an important foodborne pathogen. It is the most common pathogen of Gram-negative bacilli. It accounts for high morbidity and mortality rates worldwide [36]. In addition to food poisoning, strains of *E. coli* can cause serious illnesses such as diarrhea, peritonitis, mastitis, sepsis, pneumonia, hemorrhagic colitis, hemolytic uremic syndrome, thrombocytopenic purpura, and death. Several serotypes of *E. coli* are known to date. Most cases of hemorrhagic colitis and hemolytic uremic syndrome are linked to serotype O157:H7. This *E. coli* serotype is considered one of the most dangerous. Several epidemic of foodborne bacterial illnesses due to consumption of raw or undercooked meat contaminated with strains of STEC have been reported [37].

A single group of mucocutaneous contamination indicator germs was isolated from Gbeli samples. These are coagulase-positive *Staphylococci*. Their average load (1.3 × 10^3^ CFU/g) in the Gbeli samples is slightly above the tolerated threshold (10^3^ CFU/g). However, this average load is significantly lower than those (4 × 10^4^ to 5.2 × 10^5^ CFU/g) found by Ahmed et al. [38] in chips. Coagulase-positive *Staphylococci* in particular have become a major threat to public health due to their antibiotic resistance increase. In this bacterial group, the *Staphylococcus aureus* species particularly focuses the attention of researchers. Indeed, *S. aureus* is the common cause of food poisoning with a strong ability to tolerate a wide range of pH, temperature, and humidity. *S. aureus* produces a wide variety of heat-stable staphylococcal enterotoxins (SE) in foods responsible for staphylococcal food poisoning (SFP) in the human population. SFP is characterized by gastroenteritis and has been recognized as one of the main culprits of food poisoning epidemics worldwide [39]. Methicillin-resistant strains of *S. aureus* (MRSA) have recently been classified as high priority bacteria with the potential to cause devastating mortality globally if adequate treatment options are not developed [40]. The presence of coagulase-positive *Staphylococci* in the Gbeli remains very worrying.

**Figure 4:**
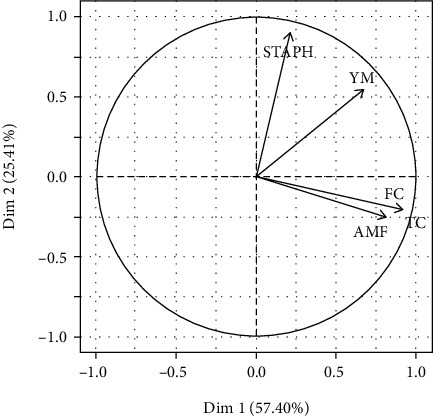
Projection of the types of microorganisms on the first two main axes.

The results of the principal component analysis indicated that the first two principal axes carry 82.81% of the starting information. This proportion is sufficient to guarantee a precision in the interpretations. Thus, Figure 4 presents the correlation between the groups of microorganisms isolated from Gbeli and these principal axes. This figure revealed that TAMF, YM, TC, and FC are strongly correlated to the first factorial axis (correlation > 0.5). This thus suggests that samples containing a significant number of TAMF also contain YM, TC, and FC in significant amounts. Moreover, YM and STAPH are strongly correlated to the second factorial axis (correlation > 0.5). This suggests that samples containing YL also contain CPS. This positive correlation relating to the presence and the load of the various microorganisms isolated from Gbeli must really be worrying. Indeed, the consumption of Gbeli therefore exposes you to a great risk of food poisoning that can be caused by several microorganisms at the same time.

## 4. Conclusion

This study provides important data on the knowledge, hygienic practices, and toxi-infectious risks associated with Gbeli. The production and sale of the Gbeli are activities carried out exclusively by adults and unschooled women. This trade generates significant income allowing these women to contribute to family expenses and to save. No major difference was noted in the production of Gbeli contrary to the sale mode. Production process, packaging form, and sale condition of Gbeli present enormous hygienic practice deficiencies. The prevalence and abundance of yeasts and molds, thermotolerant coliforms, and coagulase-positive *Staphylococci* in these chips raise huge concerns. Gbeli consumption exposes its consumers to a permanent risk of food poisoning. A real public health problem therefore arises. The administrative and health authorities must become more involved in the street food sector by investing in the control of said foods. Also, training and sensitization of street food vendors must periodically be organized on the potential dangers incurred due to their ignorance and/or negligence of good hygiene and production practices.

## Figures and Tables

**Table 1:** Sociodemographic profile of Gbeli vendors.

Parameter	Characteristic	Frequency	Percentage (*n* = 92)
Gender	Male	0	0
	Female	100	100

Age (years)	10-20	2	2.2
	20-30	14	15.2
	30-40	56	60.9
	40-50	20	21.7

Level of education	No formal education	54	58.7
	Primary school	22	23.9
	Secondary school	16	17.4

**Table 2:** Income of Gbeli woman producers.

Parameter	Characteristic	Frequency	Percentage (*n* = 92)
Unit price (USD)	0.008 0.017 0.043	00 92 00	00.0 100 00.0

Daily income (USD)	0-1.7 1.7-3.4 3.4-5.1	04 19 69	3.87 20.7 75.5

Sale order	Yes No	6 86	6.5 93.5

Home delivery	Yes No	02 90	2.2 97.8

**Table 3:** Environment, equipment, and water quality.

Parameter	Characteristic	Frequency	Percentage (*n* = 92)
Material only use for Gbeli production	No Yes	02 90	2.2 97.8

Frequency of equipment cleaning per day	One Two Three	12 80 00	13 86.9 00

Roof of production unit	Outdoor Straw Aluminum sheet Slab sheet	08 68 14 02	8.7 73.9 15.2 2.2

Cleanliness level of production unit	Unclean Clean Very clean	00 88 04	00 95.6 4.3

Water used for washing dishes and as an ingredient	Drilling water Tap water	86 06	93.5 6.5

Water treatment before use	No Yes	92 00	100 00

**Table 4:** Some recommended hygienic and manufacturing practices for Gbeli producers/vendors.

Rubrics	Proposed practices
Used water	(i) Sensitization of owners of drilling water stations on the regulatory methods of maintenance of the water system (ii) Water treatment by boiling: the water is previously filtered or decanted then simmer for about a minute (iii) Water treatment by chlorination: this consists of introducing chlorinated products (chlorine tablets, bleach, etc.) into the water to kill the microorganisms contained in it. 8 mg/L of sodium hypochlorite (NaClO) for 30 minutes makes the water potable with a NaClO residual load of 4 mg/L [32]

Production equipment	(i) Production equipment used only for the production of Gbeli (ii) Wash before and after use of the small production equipment (iii) Perform periodic maintenance of the crusher and the press

Packaging	(i) No longer use nonbiodegradable bags or cloth to cover the Gbeli donuts (ii) Make stainless steel cases and transparent glass for protecting the Gbeli donuts during sale

Use of hands	(i) Stop using bare hands (ii) Use forks or wear gloves to avoid any contamination from the vendors (iii) Wash hands after taking money from customers, because coins and banknotes can be sources of microbiological contamination (iv) Clean your hands after using your cell phone or after greeting customers or parents with your bare hands

Environment and conditions of sale	(i) Prioritize sale in stationary mode (ii) The sale of Gbeli lasts about 10 hours at room temperature. Provide a heating device (iii) Build small sheds for sale, in order to limit atmospheric contamination from air flow, dust, exhaust gases, etc.

**Table 5:** Microbiological profile of Gbeli samples.

Germs	Standard value (CFU/g)	Obtained value (CFU/g)		Signification (ANOVA)	Noncompliance rate (%)	
		Mean	SD		Specific	General
TAMF	Not defined	3.7 × 10^3^	1.1 × 10^3^	^∗∗∗^	—	
YM	Not defined	7.4 × 10^3^	1.6 × 10^3^	°	—	
TC	Not defined	3.3 × 10^2^	0.9 × 10^2^	^∗∗∗^	—	
FC	≤102	1.4 × 10^2^	0.5 × 10^2^	°	21.2	55.8
CPS	≤103	1.3 × 10^3^	0.2 × 10^3^	°	55.8	

TAMF: total mesophilic aerobic flora; YM: yeasts and molds; TC: total coliforms; FC: fecal or thermotolerant coliforms; CPS: coagulase-positive *Staphylococci*; °*p* > 0.05: not significant; ^∗^*p* < 0.05: significant; ^∗∗^*p* < 0.01: very significant; ^∗∗∗^*p* < 0.001: very highly significant; CFU/g: colony forming unit per gram of sample; SD: standard deviation.

## Data Availability

The datasets during the current study are available from the corresponding author on reasonable request.
